# Comparative performance of MRI-derived PRECISE scores and delta-radiomics models for the prediction of prostate cancer progression in patients on active surveillance

**DOI:** 10.1007/s00330-021-08151-x

**Published:** 2021-07-13

**Authors:** Nikita Sushentsev, Leonardo Rundo, Oleg Blyuss, Tatiana Nazarenko, Aleksandr Suvorov, Vincent J Gnanapragasam, Evis Sala, Tristan Barrett

**Affiliations:** 1 Department of Radiology, Addenbrooke’s Hospital and University of Cambridge, Cambridge, UK; 2 Department of Radiology, University of Cambridge School of Clinical Medicine, Box 218, Cambridge Biomedical Campus, Cambridge, CB2 0QQ UK; 3 Cancer Research UK Cambridge Centre, University of Cambridge, Cambridge, UK; 4 School of Physics, Engineering & Computer Science, University of Hertfordshire, Hatfield, UK; 5 Department of Paediatrics and Paediatric Infectious Diseases, Sechenov First Moscow State Medical University, Moscow, Russia; 6 Department of Applied Mathematics, Lobachevsky State University of Nizhny Novgorod, Nizhny Novgorod, Russia; 7 Department of Mathematics and Institute for Women’s Health, University College London, London, UK; 8 World-Class Research Center “Digital Biodesign and Personalised Healthcare”, Sechenov First Moscow State Medical University, Moscow, Russia; 9 Division of Urology, Department of Surgery, University of Cambridge, Cambridge, UK; 10 Cambridge Urology Translational Research and Clinical Trials Office, University of Cambridge, Cambridge, UK

**Keywords:** Prostate cancer, Magnetic resonance imaging, Active surveillance, PRECISE, Machine learning

## Abstract

**Objectives:**

To compare the performance of the PRECISE scoring system against several MRI-derived delta-radiomics models for predicting histopathological prostate cancer (PCa) progression in patients on active surveillance (AS).

**Methods:**

The study included AS patients with biopsy-proven PCa with a minimum follow-up of 2 years and at least one repeat targeted biopsy. Histopathological progression was defined as grade group progression from diagnostic biopsy. The control group included patients with both radiologically and histopathologically stable disease. PRECISE scores were applied prospectively by four uro-radiologists with 5–16 years’ experience. T2WI- and ADC-derived delta-radiomics features were computed using baseline and latest available MRI scans, with the predictive modelling performed using the parenclitic networks (PN), least absolute shrinkage and selection operator (LASSO) logistic regression, and random forests (RF) algorithms. Standard measures of discrimination and areas under the ROC curve (AUCs) were calculated, with AUCs compared using DeLong’s test.

**Results:**

The study included 64 patients (27 progressors and 37 non-progressors) with a median follow-up of 46 months. PRECISE scores had the highest specificity (94.7%) and positive predictive value (90.9%), whilst RF had the highest sensitivity (92.6%) and negative predictive value (92.6%) for predicting disease progression. The AUC for PRECISE (84.4%) was non-significantly higher than AUCs of 81.5%, 78.0%, and 80.9% for PN, LASSO regression, and RF, respectively (*p* = 0.64, 0.43, and 0.57, respectively). No significant differences were observed between AUCs of the three delta-radiomics models (p-value range 0.34–0.77).

**Conclusions:**

PRECISE and delta-radiomics models achieved comparably good performance for predicting PCa progression in AS patients.

**Key Points:**

• *The observed high specificity and PPV of PRECISE are complemented by the high sensitivity and NPV of delta-radiomics, suggesting a possible synergy between the two image assessment approaches.*

• *The comparable performance of delta-radiomics to PRECISE scores applied by expert readers highlights the prospective use of the former as an objective and standardisable quantitative tool for MRI-guided AS follow-up.*

*• The marginally superior performance of parenclitic networks compared to conventional machine learning algorithms warrants its further use in radiomics research.*

**Supplementary Information:**

The online version contains supplementary material available at 10.1007/s00330-021-08151-x.

## Introduction

Prostate cancer (PCa) is the second commonest and the fifth deadliest male cancer worldwide [1]. In the USA and the UK, nearly half of men present with low- and intermediate-risk localised disease [2, 3], for which active surveillance (AS) is the recommended management option [4–6]. Simultaneously, a cumulative 5-year AS dropout rate due to disease progression is 27% [7], which is partially driven by the lack of consensus on AS protocols and the definition of disease progression across guidelines and individual centres [8–11]. Whilst tumour progression is the expected natural outcome of AS, there is a lack of objective non-invasive diagnostic tools enabling continuous re-evaluation of the risk of PCa progression. If proven accurate and standardisable, such tools could help clinicians make more informed decisions on the need for switching to radical treatment without repeat biopsies, thereby reducing the risk of associated complications and costs to healthcare systems.

At this stage, this unmet clinical need is partially addressed by the increasing reliance on magnetic resonance imaging (MRI) as an integral part of AS follow-up [12–15]. The PRECISE scoring system was developed in 2017 [16] in order to standardise reporting of serial MRI scans in patients on AS to detect clinically significant radiological changes sufficient to either trigger additional investigations or switch to immediate radical treatment. Since then, several groups [17–21] have demonstrated a high negative predictive value of PRECISE, suggesting that it can be used to avoid routine repeat biopsy if the disease is regarded as radiologically stable. However, the comparatively moderate positive predictive value of PRECISE for predicting histopathological disease progression emphasises the relatively subjective nature of the system, particularly when determining PRECISE category 4 lesions as demonstrating “clinically significant” radiological progression [12]. Furthermore, PRECISE has been validated exclusively by expert readers in academic centres, which may limit its generalisability and lead to a reduced performance when used by non-expert radiologists.

Quantitative imaging techniques may prove clinically useful by providing more objective and expertise-independent measures of the underlying biological changes occurring over the course of PCa natural history on AS. One such technique is delta-radiomics, which analyses changes in MRI-derived texture features obtained at two time points [22]. In the AS setting, delta-radiomic features may be compared between any two consecutive MRI scans obtained from the same patient to provide a quantitative readout of the underlying histological changes within a lesion of interest. Therefore, in this proof-of-concept study, we compared the diagnostic performance of PRECISE applied by expert readers against several MRI-derived delta-radiomics models for the purpose of predicting histopathological PCa progression in patients enrolled on AS.

## Methods

### Patient population

The local institutional review board (NRES Committee East of England, UK) waived the need for informed consent for retrospective data analysis obtained as part of a service evaluation of the prostate diagnostic pathway. The study included consecutive patients with biopsy-proven PCa enrolled on the local AS programme with a minimum follow-up of 2 years, with their first and last 3T MRI scans performed on the same magnet, and at least one repeat targeted biopsy performed within a year of the last MRI. The exclusion criteria were the absence of MR-visible lesions, prior or interim treatment for PCa or benign disease, and the presence of total hip replacement or other pelvic metalwork. A total of 281 patients enrolled on AS in our centre between November 2012 and November 2018 were screened, of whom 217 were excluded.

The remaining 64 patients were divided into two groups depending on their disease progression status. AS progression (n = 27) was defined as a switch to radical treatment prompted by confirmed histopathological progression on repeat targeted biopsy (grade group progression from diagnostic biopsy). The control group (n = 37) included patients whose disease remained both radiologically (PRECISE score 3 [16]) and histopathologically stable over the course of AS.

### MRI acquisition parameters

Patients underwent prostate MRI on a 3-T MR750 scanner (GE Healthcare) using a 32-channel receiver coil. Unless clinically contraindicated, intravenous injection of hyoscine butylbromide (Buscopan, 20 mg/mL; Boehringer) was administered prior to imaging to reduce peristaltic movement [23]; no additional patient preparation measures were taken. Multiparametric MRI protocol included axial T1, multiplanar high-resolution T2-weighted 2D fast recovery FSE, spin-echo echo-planar imaging pulse DWI, and dynamic contrast enhancement imaging, with acquisition parameters summarised in Supplementary Table S1.

### Biopsy technique

Depending on clinical recommendation, either transrectal (DynaCAD, InVivo Corp) or transperineal (Biopsee, Oncology Systems Limited) biopsies were performed by three urologists with 8–20 years’ experience using MRI/ultrasound fusion. Twelve systematic cores were taken as part of the transrectal approach, and 24 systematic cores were obtained during transperineal procedures, following the Ginsburg protocol [24]. In addition, 2–4 separate target cores corresponding to lesions outlined on MRI were sampled, as previously described [25]. Repeat targeted biopsies were performed at time points specified by the local protocol (12 and 36 months) if not triggered earlier by clinical suspicion of progression, encompassing either three consecutive elevated PSA levels above the pre-defined threshold or suspected radiological progression (PRECISE scores 4–5). At baseline, 13 and 51 biopsies were performed using transrectal and transperineal approaches, respectively. At follow-up, 18 and 46 biopsies were performed using transrectal and transperineal approaches, respectively.

### Image segmentation and analysis

**Fig. 1:**
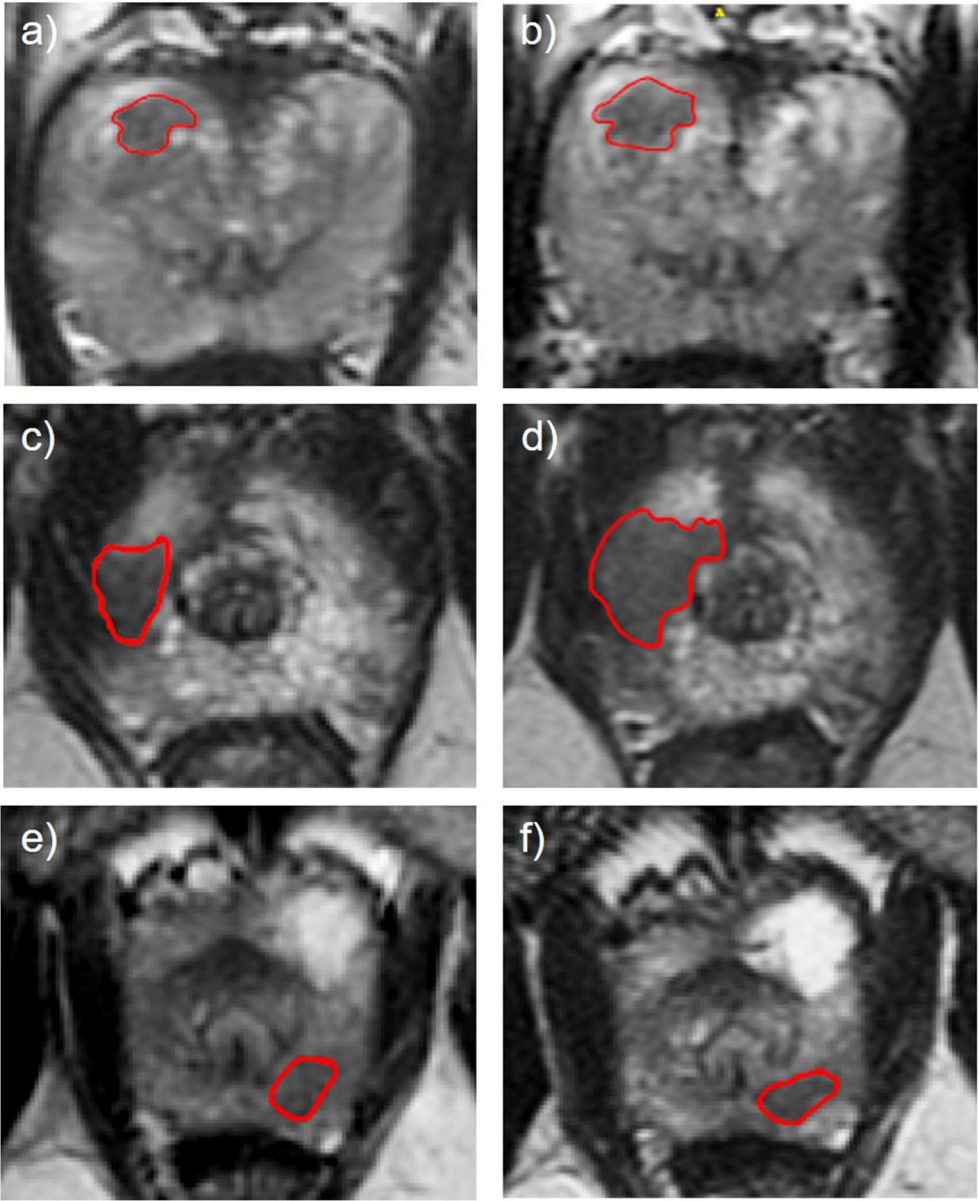
Comparison of T2-weighted images of the prostate obtained at baseline pre-biopsy (**a**, **c**, **e**) and follow-up (**b**, **d**, **f**) MRI scans from patients enrolled on active surveillance. Images (**a**, **b**) were obtained from a patient with stable 3 + 3 = 6 disease that showed neither radiological nor histopathological progression over a follow-up period of 3 years (PRECISE 3). Images (**c**, **d**) were obtained from a patient with both radiological (PRECISE 5) and histopathological (3 + 3 = 6 to 4 + 3 = 7) progression. Images (**e**, **f**) were obtained from a patient with confirmed histopathological progression (3 + 3 = 6 to 3 + 4 = 7) but radiologically stable disease (PRECISE 3). In all presented cases, the clinical outcome was successfully predicted by all three delta-radiomics models used

Tumour ROIs were drawn on anatomical T2WI (Fig. 1) and ADC maps by a fellowship-trained uro-radiologist (T.B.) with 13 years’ experience of reporting prostate MRI and an imaging research fellow (N.S.) with 4 years’ experience. The segmentation was performed in consensus using the open-source software ITK-SNAP [26], with all cases outlined jointly by the two readers. The reliability of image segmentation by readers was evaluated by applying ROI morphological perturbations using the “Scipy.ndimage.morphology” functions (binary_opening and binary_closing) in the SciPy version 1.3.2 multi-dimensional image processing package.

Follow-up MRI studies were scored on a 5-point PRECISE scale described in Supplementary Table S2 [16]. PRECISE scores were applied prospectively by four sub-specialist uro-radiologists with 5–16 years’ experience of reporting prostate MRI, with each having read > 2,000 cases and considered to be experts [27, 28]. At the time of reporting, the readers were not blinded to clinical information, including PSA and PSA density dynamics. For the purposes of predictive modelling, the PRECISE scores were dichotomised at a cutoff value of 4 since values 1–3 and 4–5 indicate resolution of previous features/stable disease and radiological disease progression, respectively.

### Delta-radiomics analysis

**Fig. 2:**
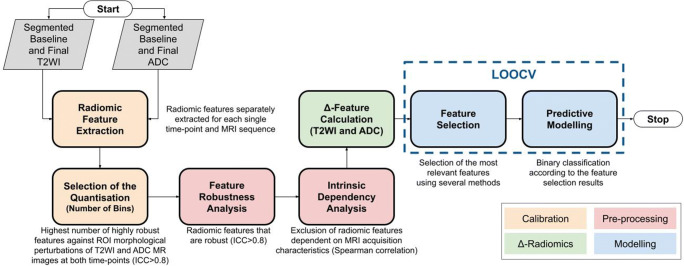
Flow diagram summarising the key stages of delta-radiomics analysis used in this study, including calibration, pre-processing, delta-radiomics feature calculation, and predictive modelling using the leave-one-out cross-validation (LOOCV) approach. ADC, apparent diffusion coefficient; ICC, intraclass correlation coefficient; MRI, magnetic resonance imaging; T2WI, T2-weighted imaging

The overall workflow of the radiomics pipeline utilised to develop and validate predictive models for PCa progression on AS is illustrated in Fig. 2. The T2WI- and ADC-derived texture features (summarised in Supplementary Table S3**)** were extracted using PyRadiomics version 2.0 and Python version 3.7.5 [29, 30]. 3D feature computation without any resampling was used to avoid interpolation artifacts. According to the Imaging Biomarker Standardisation Initiative (IBSI) [30], the use of the number of bins is favoured over the bin width in the case of arbitrary intensity units, such as MRI. Hence, no re-segmentation (i.e. the voxels outside a specified range being removed from the mask prior to texture feature calculation) was applied.

Feature robustness was assessed by applying ROI morphological perturbations and evaluating the relationship between individual features and MRI acquisition parameters [31–33] as described in detail in the *Calibration and pre-processing* section of the Electronic Supplementary Material. This procedure also simulates the ROI variability by considering intra- and inter-reader dependence during manual image segmentation [34]. Only features considered robust at both time points were included in the delta-radiomics predictive modelling.

### Delta-radiomics predictive modelling

The delta-radiomics features were computed as the arithmetic difference between the final and baseline features. For each radiomic feature (separately for T2WI and ADC), let *f*_base_ and *f*_final_ be its value at the baseline and final scan, respectively, Δ*f* was calculated as:

$$ \Delta f={f}_{\mathrm{final}}-{f}_{\mathrm{base}}. $$

Predictive modelling was then carried out using three machine learning methods: parenclitic networks [35], least absolute shrinkage and selection operator (LASSO) logistic regression [36], and random forests [37]. For the latter, we used 500 trees with a maximum depth of 10 (for reproducibility, a random_state parameter of 42 was set). The binary outcome of PCa progression was used as an outcome, with all delta-radiomics features used as predictors. The predictive performance of each feature was assessed using a leave-one-out cross-validation (LOOCV) scheme. The prediction was made for each sample by excluding (withholding) it from the dataset, training the classifier on the remaining (independent) samples, and then generating predictions for the withheld samples using the trained model.

### Statistical and computational analysis

Normal distribution of the data was assessed using the D’Agostino-Pearson test (threshold *p* ≥ 0.05). Intergroup comparison of patient age, PSA, gland volume, PSA density, and AS follow-up length was performed using the Mann-Whitney U test. The relationship between the radiomic feature and individual MRI acquisition parameters was assessed using Spearman’s correlation analysis.

Random forests and LASSO regression were implemented in Python 3.9.0 using the pandas version 1.2.4, scikit-learn version 0.24.2, glmnet_python version 1.0, and rpy2 version 3.4.2 modules. In parenclitic networks, a support vector machine (SVM) with radial basis functions was trained for each pair of features using the “e1071” R package with default settings. For each sample, a network was then built wherein vertices corresponded to features and the edge weight was the disease progression probability as predicted by the SVM classifier. The mean of the vertices degrees was calculated, and a generalised linear model (GLM) classification was then constructed using the “stats” R package with default settings. The performance of PRECISE-based and delta-radiomics-based predictive models was assessed with measures of discrimination. Specificity and sensitivity were derived using non-parametric stratified resampling with the percentile method (2,000 bootstrap replicates) [38]. In addition, areas under the ROC curve (AUCs) were calculated for each model, alongside 95% confidence intervals using DeLong’s asymptomatically exact method to evaluate the uncertainty of each AUC [39]. AUC values were compared between the models using DeLong’s test for correlated/paired AUCs [39]. Positive and negative predictive values (PPV and NPV) and their 95% confidence intervals were computed using the standard approach [40]. Statistical analysis was performed in R version 3.5.1 (R Foundation for Statistical Computing) using the “pROC” and “reportROC” packages.

## Results

### Patient characteristics

The study included 64 patients enrolled on the AS programme in our centre between May 2013 and May 2018. The key clinicopathological characteristics of the patient cohort are summarised in Table 1. Twenty-seven patients showed histopathological disease progression and 37 patients harboured stable disease. Non-progressors were followed up for a median time of 47 months (IQR, 44–60 months), and time to progression in progressors was significantly shorter at 43 months (IQR, 24–29 months; *p* = 0.009). PSA and PSA density were significantly higher in progressors compared to non-progressors (*p* = 0.01 and *p* = 0.002, respectively), whilst age, gland volume, and PI-RADS scores were similar and demonstrated no significant difference between the two groups (Table 1). At enrollment biopsy, 51/65 (79%) and 14/65 (21%) lesions represented ISUP grade group 1 and 2 tumours, respectively, with 46/65 (71%) and 19/65 (29%) target lesions located in the peripheral and transition zones, respectively.

**Table 1:** Summary baseline clinicopathological characteristics of the study cohort. The *p*-values are presented for an intergroup comparison between progressors and non-progressors performed using the Mann-Whitney U test. *AS*, active surveillance; *PSA*, prostate-specific antigen

Parameter	Total cohort (n = 64)	Progressors (n = 27)	Non-progressors (n = 37)	*p*-value
Age, years	67 (60–69)	66 (60–69)	67 (61–69)	0.9218
PSA, ng/mL	5.6 (3.6–7.7)	7.0 (5.2–8.7)	5.0 (3.17–6.9)	0.0102
Gland volume, mL	45.0 (33.0–63.8)	45.0 (29.0–52.0)	45.0 (36.6–66.2)	0.1851
PSA density	0.11 (0.08–0.19)	0.17 (0.11–0.27)	0.09 (0.06–0.16)	0.0024
AS follow-up, mo	46 (35–52)	43 (24–49)	47 (44–60)	0.0093
Biopsy grade group 1 (3 + 3 = 6), n	51 (78%)	23 (85%)	28 (74%)	-
Biopsy grade group 2 (3 + 4 = 7), n	14 (22%)	4 (15%)	10 (26%)	
Target lesion in the peripheral zone, n	46 (70%)	17 (63%)	29 (76%)	-
Target lesion in the transition zone, n	19 (30%)	10 (27%)	9 (24%)	

### Delta-radiomics analysis: calibration and pre-processing

The numbers of highly robust features per number of bins identified at the calibration and pre-processing stage are presented in Table 2. Spearman’s correlation analysis showed no significant relationship between any of the texture features and MRI acquisition parameters (*p* > 0.05 for all with no multiplicity correction applied). The selected number of bins was, therefore, 128, with 34 T2WI- and 53 ADC-derived texture features overlapping at both time points (**Supplementary Table S4**).

**Table 2:** The number of features with high robustness (ICC > 0.8) by varying the number of bins in the quantisation step for radiomic feature extraction for T2-weighted and ADC MR images at both baseline and final time points. *ADC*, apparent diffusion coefficient; *T2WI*, T2-weighted imaging

Number of bins	Number of features with high robustness				
	Baseline		Final		Total
	T2WI	ADC	T2WI	ADC	
8	38	47	67	47	199
16	34	49	67	51	201
32	32	52	63	54	201
64	33	55	64	54	206
128	34	57	69	54	214
256	33	56	70	55	214

### PRECISE versus delta-radiomics for predicting disease progression

**Fig. 3:**
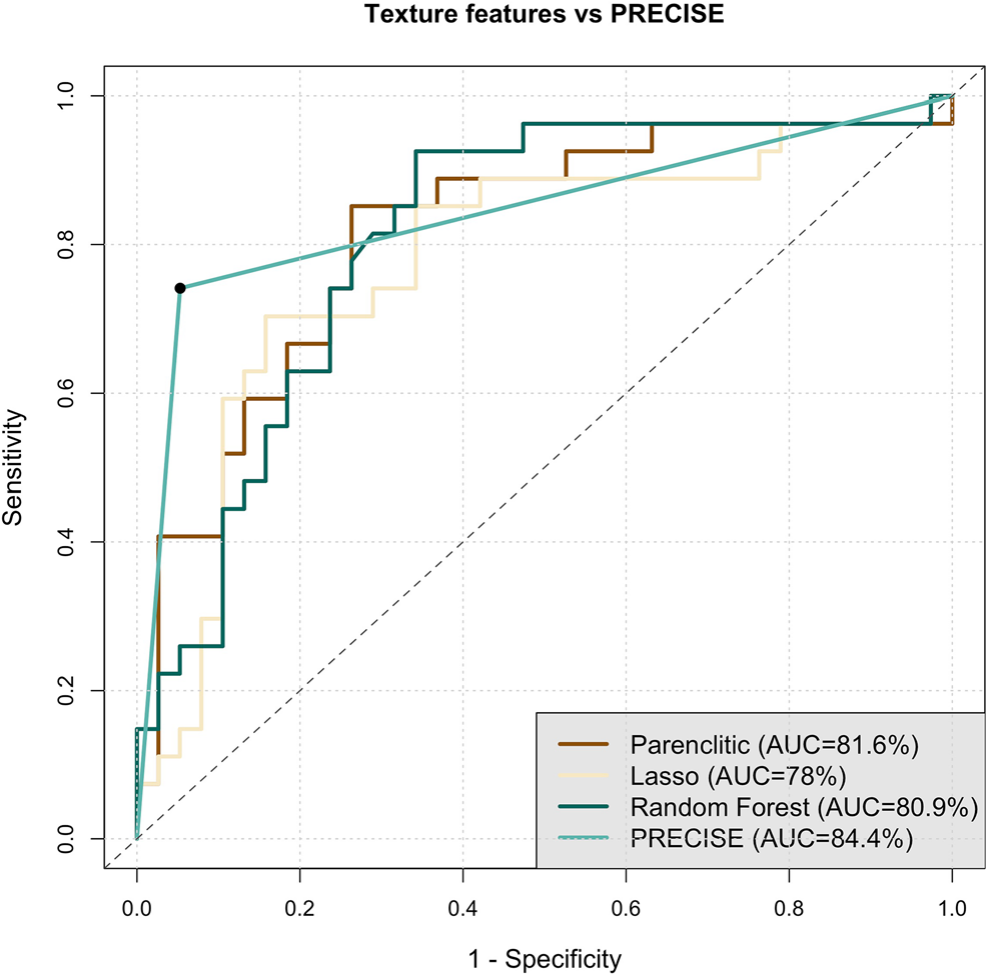
Receiver operating characteristic (ROC) curves for PRECISE, parenclitic networks, lasso regression, and random forest for predicting histopathological progression of prostate cancer in patients on active surveillance. The embedded legend denotes areas under ROC curves for each method

Table 3 presents summary performance characteristics of PRECISE and the three machine learning approaches: parenclitic networks, LASSO regression, and random forests. The analysis of sensitivity, specificity, PPV, and NPV did not allow to identify the best-performing approach: PRECISE score had the highest specificity (94.7%) and PPV (90.9%), whilst random forests had the highest sensitivity (92.6%) and NPV (92.6%). AUC for PRECISE (84.4%) was the highest of all, compared to 81.5%, 78.0%, and 80.9% for parenclitic networks, LASSO regression, and random forests, respectively (Fig. 3). This difference, however, was non-significant: *p* = 0.643 for PRECISE versus parenclitic networks, *p* = 0.342 for PRECISE versus LASSO regression, and *p* = 0.572 for PRECISE versus random forests. No significant differences were reported between AUCs of the three delta-radiomics models (*p*-value range 0.342–0.768).

**Table 3:** Summary performance characteristics of PRECISE, alongside parenclitic networks, LASSO regression, and random forests delta-radiomics models for predicting histopathological progression of prostate cancer in patients on active surveillance. *AUC*, area under the receiver operator characteristic curve; *LASSO*, least absolute shrinkage and selection operator; *NPV*, negative predictive value; *PPV*, positive predictive value; *PRECISE*, Prostate Cancer Radiological Estimation of Change in Sequential Evaluation

Method	Sensitivity	Specificity	PPV	NPV	AUC
PRECISE	74.1 (57.5–90.6)	94.7 (87.6–1)	90.9 (78.9–1)	83.7 (72.7–94.8)	84.4 (72.6–96.2)
Parenclitic networks	85.2 (71.8–98.6)	73.7 (59.7–87.7)	69.7 (54–85.4)	87.5 (76–99)	81.6 (70.6–92.5)
LASSO regression	70.4 (53.1–87.6)	84.2 (72.6–95.8)	76.0 (59.3–92.7)	80.0 (67.6–92.4)	78.0 (65.8–90.1)
Random forests	92.6 (82.7–1)	65.8 (50.7–80.9)	65.8 (50.7–80.9)	92.6 (82.7–1)	80.9 (70–91.9)

## Discussion

This proof-of-concept study investigates the comparative performance of the MRI-derived PRECISE scoring system applied in routine clinical practice by expert uro-radiologists versus three delta-radiomics models developed using the parenclitic networks, LASSO regression, and random forests machine learning methods. The primary performance metric was the ability to predict histopathological progression of PCa in patients enrolled on AS. Although PRECISE performance characteristics appeared marginally superior, there was no significance difference between the AUCs achieved by all methods used in this study. These results pave the way for future investigations evaluating the role of MRI-derived delta-radiomics as an alternative quantitative means of MRI-guided follow-up of AS patients that could be standardisable and less dependent on reader experience.

In this study, PRECISE scoring demonstrated a high performance for predicting PCa histopathological progression with an AUC of 0.84, which is in line with AUCs of 0.83 and 0.82 reported by Dieffenbacher et al [17] and Caglic et al [21], respectively. It should be noted that, in addition to the readers’ expertise, the availability of clinical parameters may help supplement clinical decision-making and further improve performance, for example knowledge of PSA kinetics may help call PRECISE 4, when features of radiological progression may be otherwise equivocal. Among the machine learning techniques, random forests showed the best performance with the highest sensitivity (92.6%) and NPV (92.6%).

To our knowledge, this is the first study applying delta-radiomics in the setting of MRI follow-up in PCa AS. The comparable performance of standalone delta-radiomics models, which only account for quantitative image-derived texture features of the disease and were subject to rigorous cross-validation to reduce overfitting, offers potential for future studies attempting to introduce accurate and objective tools for evaluating PCa radiological progression on AS. Previous studies in PCa using MRI-derived delta-radiomics to predict tumour response to radiotherapy [41, 42] have also shown promising results. In other tumour types, delta-radiomics studies have likewise focused on predicting treatment response, including lung [22, 43], rectal [44, 45], and gastric [46] cancers, as well as bone and soft tissue malignancies [47, 48]. With the concept of AS being relatively unique to PCa, using delta-radiomics to track intratumoural morphological changes occurring naturally in the absence of any external interventions may offer insights into the biological significance of certain texture features that show strong correlation with the underlying cytoarchitectural patterns; this technique may additionally be applicable to other tumour types.

In this study, we were the first to apply the parenclitic networks for delta-radiomics predictive modelling. The particular strength of this novel machine learning approach is that it is deemed useful in a setting where the number of features is large whilst the sample size is limited [49], which is often the case with radiomics studies. Another strength of parenclitic networks is the ability to embed any multivariate data into the low-dimensional space of topological indices, which avoids the risk of overfitting. In this study, no difference in performance was observed between any of the machine learning algorithms used. The marginally better performance of the clinically applied PRECISE system may be explained by the use of stringent and unbiased strategies to reduce overfitting in the delta-radiomics modelling. These proof-of-concept results, therefore, offer promise for the development of improved delta-radiomics performance in larger cohorts.

Our study has several limitations. The sample size was relatively small, which was dictated by the stringent inclusion criteria requiring the presence of MR-visible lesions, which represents only around 50% of patients in AS cohorts [21] and mandating repeat targeted biopsies performed within a year of the most recent MRI, which was necessary for histopathological confirmation of progression. Gaining access to a larger cohort will also enable us to fit a single parenclitic networks model and fine-tune the hyperparameters for the SVM. Furthermore, the lesion-centred delta-radiomics approach was selected for several reasons. Firstly, the presence of MR-visible lesions is associated with an increased risk of progression on AS [21], thereby warranting closer follow-up of such patients. Secondly, as this is the first study to use delta-radiomics in this setting, focusing on the analysis of exclusively intratumoural rather than whole-gland texture features was deliberate in order to reduce the complexity of the analysis. The use of a whole-gland image segmentation approach [50, 51] coupled with MRI-derived habitat radiomics [52, 53] is an area for future work and would be applicable to all patients enrolled on AS. This will, however, require even more stringent pre-processing, calibration, modelling, and cross-validation steps to ensure that the resulting predictive models are not overfitted by features possibly arising from temporal changes in concurrent benign conditions (such as benign prostatic hyperplasia or prostatitis). Finally, as discussed previously, the reporting clinicians were not prospectively blinded to clinical information, which may have artificially increased the performance of the PRECISE scoring system. However, benchmarking alternative diagnostic modalities against the real-life performance of those already applied in routine clinical practice offers the fastest route to understanding the feasibility of their clinical translation.

In conclusion, MRI-derived delta-radiomics demonstrated comparable performance to expert prostate MRI readers and offer an objective and standardisable method for predicting histopathological progression of PCa in patients enrolled on AS programmes. These results pave the way for future multicentre studies investigating the diagnostic utility of more complex predictive models incorporating delta-radiomics features alongside standard-of-care clinicopathological predictors of PCa progression across different centres.

## Supplementary Information

ESM 1 (DOCX 1238 kb)

## Abbreviations

ADC: Apparent diffusion coefficient
AS: Active surveillance
AUC: Area under the receiver operator curve
LASSO: Least absolute shrinkage and selection operator
LOOCV: Leave-one-out cross-validation
MRI: Magnetic resonance imaging
NPV: Negative predictive value
PCa: Prostate cancer
PN: Parenclitic networks
PPV: Positive predictive value
PRECISE: Prostate Cancer Radiological Estimation of Change in Sequential Evaluation
PSA: Prostate-specific antigen
RF: Random forests
ROC: Receiver operator curve
T2WI: T2-weighted imaging

## Declarations

### Guarantor

The scientific guarantor of this publication is Dr Tristan Barrett.

### Statistics and biometry

Four of the authors have significant statistical expertise (OB, TN, AS, LR).

### Study subjects or cohorts overlap

Some study subjects or cohorts have been previously reported in:

Caglic I, Sushentsev N, Gnanapragasam V, Sala E, Shaida N, Koo B, et al. MRI-derived PRECISE scores for predicting pathologically-confirmed radiological progression in prostate cancer patients on active surveillance. *Eur Radiol* 2020. 2020; 1–10. doi:10.1007/s00330-020-07336-0.

### Methodology

• Retrospective

• Observational

• Performed at one institution
