# Supplementary material for: Comparative performance of MRI-derived PRECISE scores and delta-radiomics models for the prediction of prostate cancer progression in patients on active surveillance
Source: Eur Radiol. 2021 Jul 13;32(1):680–9. doi: 10.1007/s00330-021-08151-x (PMC8660717; doi:10.1007/s00330-021-08151-x)
Supplement: Supplementary file 1 — (DOCX 1238 kb) [file 330_2021_8151_MOESM1_ESM.docx]

| **Parameter** | **Localizer** | **Axial T1 FSE** | **Axial T2 FSE** | **Sagittal T2 FSE** | **Axial DWI** | **Axial DWI Focus** | **DCE LAVA*** |
| --- | --- | --- | --- | --- | --- | --- | --- |
| TE/TR, ms | 20/200 | 30/789 | 102/3743 | 102/3743 | 85/3775 | 60/4000 | min full/4.3 |
| FOV, cm | 30 | 32 | 18 | 22 | 28 | 24 | 24 |
| Matrix | 256 | 512 | 384 | 288 | 128 | 356 | 192 |
| Slice thickness, mm | 3-4 | 6 | 3 | 2 | 3 | 3 | 3 |
| Gap, mm | 0 | 2 | 0 | 0 | 0 | 0 | 0 |
| Phase | 128 | 320 | 224 | 224 | 128 | 80 | 192 |
| b-values, s/mm^2^ | - | - | - | - | 100, 750, 1400 | 100, 2000 | - |
| Synthetic b-values, s/mm^2^ | - | - | - | - | 2000 | 2500 | - |

**Comparative performance of MRI-derived PRECISE scores and delta-radiomics models for the prediction of prostate cancer progression in patients on active surveillance**

**Supplementary Table S1.** Multiparametric prostate MRI protocol used in this study. TR = repetition time, TE = echo time, FOV = field of view, DCE = dynamic contrast enhancement, FSE = fast spin echo, DWI = diffusion-weighted imaging, LAVA = liver acquisition with volume acceleration. *DCE-MRI was only performed at baseline and not in follow-up scans; a bolus of Gadobutrol was used (Gadovist, 0.1 mmol/kg; Bayer) at 28 seconds via a power injector, at a rate of 3 mL/s (dose 0.1 mmol/kg).

| **PRECISE score** | **Likelihood of radiological progression** |
| --- | --- |
| 1 | Resolution of previous features suspicious on MRI |
| 2 | Reduction in volume and/or conspicuity of features suspicious for prostate cancer |
| 3 | Stable MRI appearance: no new focal/diffuse lesions |
| 4 | Increase in size and/or conspicuity of features suspicious for prostate cancer |
| 5 | Definite radiologic stage progression (ECE, SV involvement, LN involvement, metastasis) |

**Supplementary Table S2.** PRECISE scoring system (adapted from [1]).


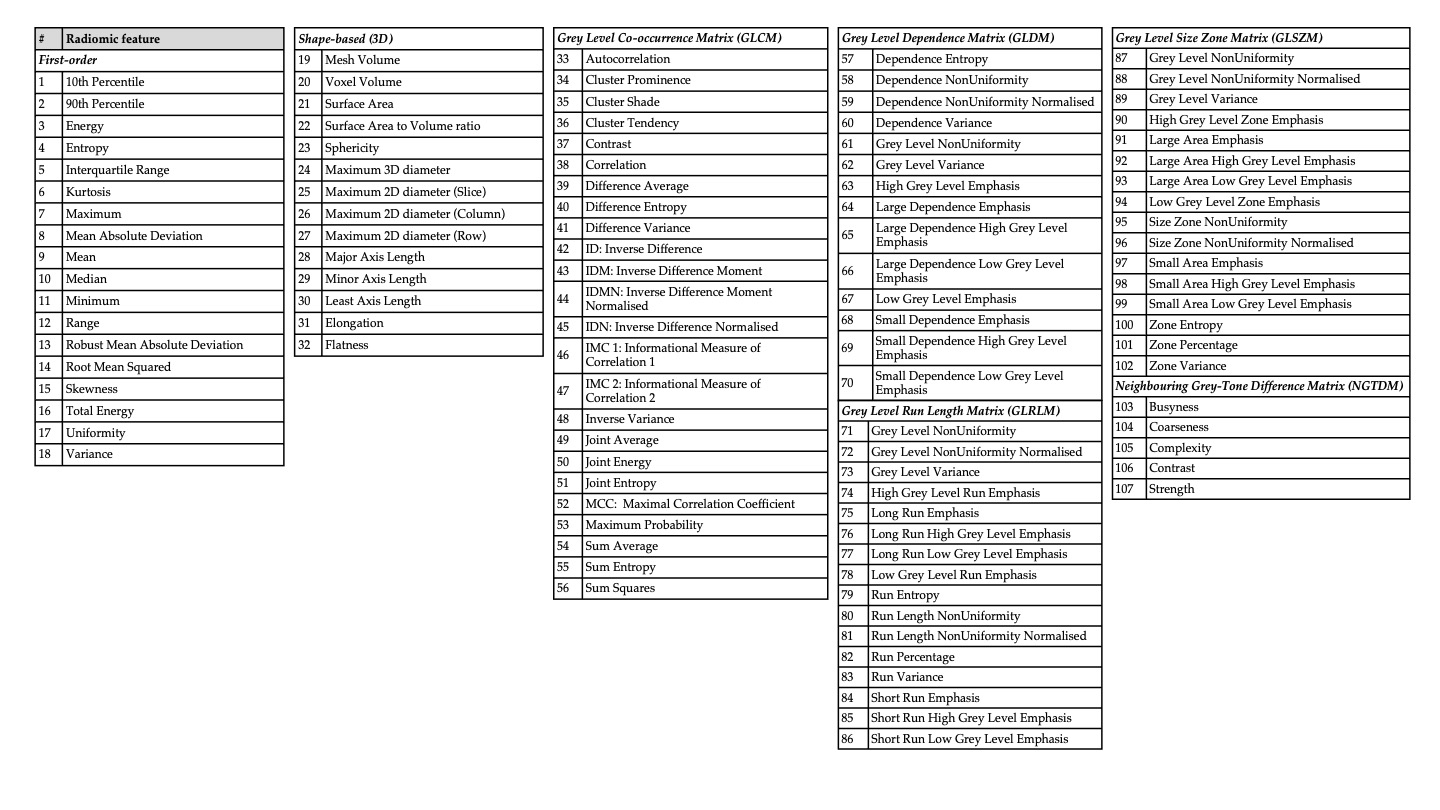


**Supplementary Table S3.** Radiomic features extracted from the ROIs in this study. Feature description can be found in the online PyRadiomics documentation (<https://pyradiomics.readthedocs.io/en/latest/>).

**Delta-radiomics analysis**

*Calibration and pre-processing*

The extracted features were considered highly robust if they remained unchanged following the application of ROI perturbations and were independent from the MRI acquisition parameters.

T2WI- and ADC-derived ROIs were perturbed by using morphological operators (i.e. opening and closing with a 3D spherical structuring element of 1-pixel radius). We thereby produced three versions of each ROI: original, opening, and closing. This procedure tests the variability of ROIs by emulating the intra- and inter-reader dependence of manual contouring [2]. Starting from these three sets of ROIs, the radiomic features were extracted for both time-points and MRI sequences using different quantisation configurations: the number of bins varied in {8, 16, 32, 64, 128, 256}. Thus, the two perturbations applied to the original ROIs and the different quantization settings yielded 18 configurations of radiomic features for each sequence and time-point pair.

The ICC was considered to determine the most robust features against the ROI perturbations whilst the number of bins varied too [3]. ICC analysis was applied to these 18 configurations of features for identifying the number of bins that achieved the largest set of highly robust features extracted separately on T2WI and ADC. In particular, we considered the two-way random-effects model (or mixed-effects), consistency, single rater/measurement, *ICC*(3,1) [4]:

| $ICC(3,1) =\frac{{MS}_{R}- {MS}_{E}}{{MS}_{R} + (k-1) {MS}_{E}}$, |  |
| --- | --- |

where ${MS}_{R}$ and ${MS}_{E}$ are the mean square for rows and mean square for error, respectively. The cutoff value was set to 0.8.

The chosen number of bins represents the most reliable quantisation configuration (i.e. rebinning) according to the ROI perturbations *via* morphological operators. A cut-off value of 0.8 was used for the ICC to identify the number of features with high robustness. The used quantisation configuration was selected by considering the number of bins that obtained the highest number of robust features for T2w and ADC at both time-points. The highly robust features with ICC > 0.8 were then used in the downstream pre-processing phases.

Secondly, the extracted features might be affected by the MRI acquisition characteristics, such as scanner type, scanner settings, imaging protocols and acquisition parameters [4]. In this study, we calculated the Spearman correlation coefficient for each radiomic feature against the following MRI acquisition parameters: (*i*) echo time (TE); (*ii*) repetition time (TR); (*iii*) flip angle; (*iv*) slice thickness; (*v*) spacing between slices; (*vi*) pixel spacing.

Only features considered robust at both time-points were included in the delta-radiomics predictive modelling.

**T2WI: ADC:**


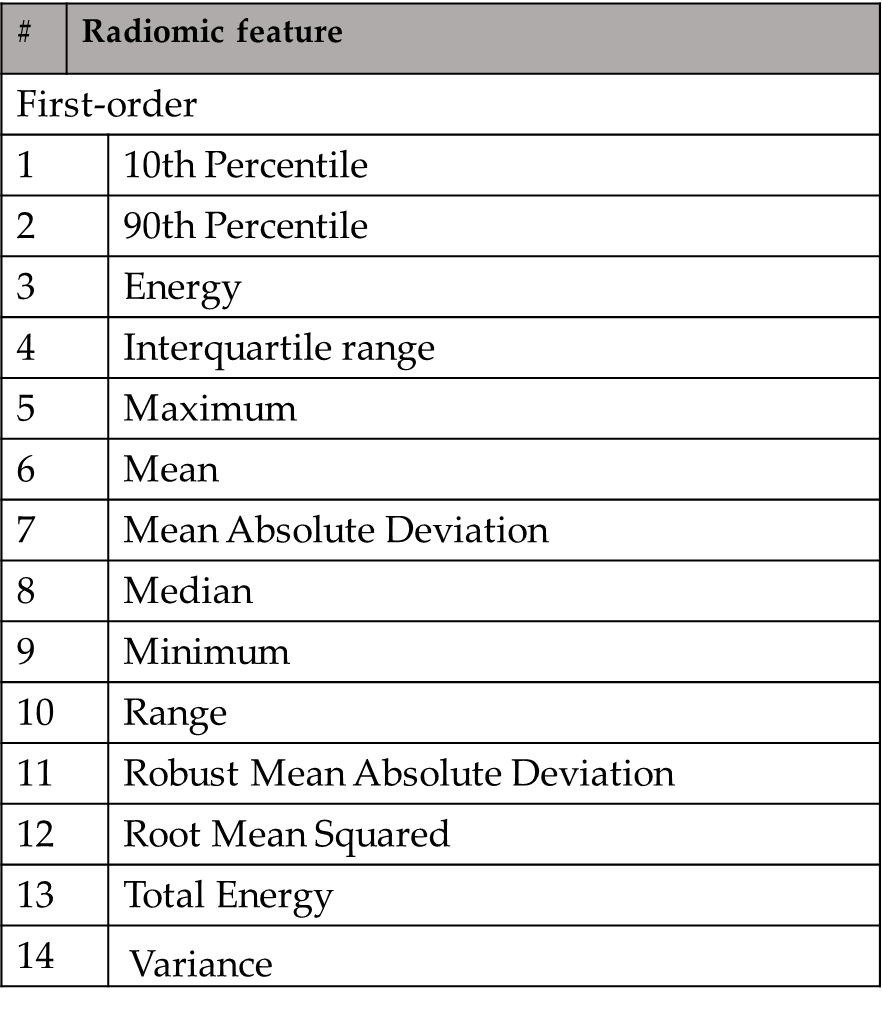

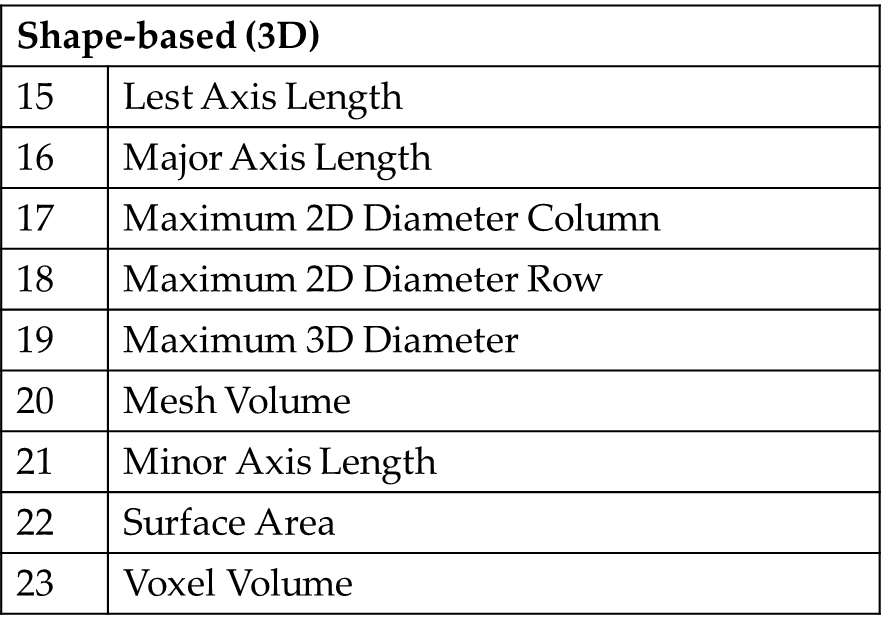

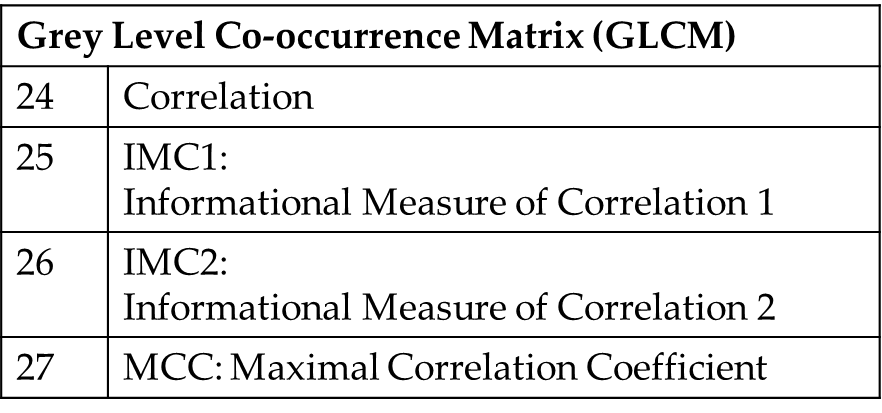

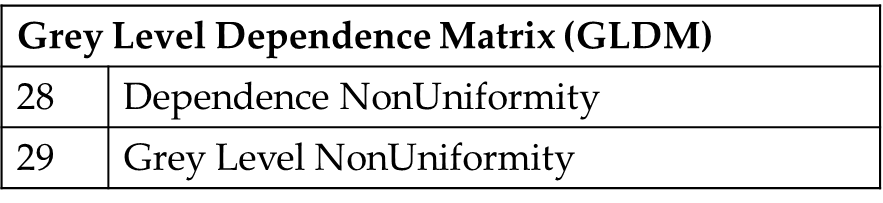

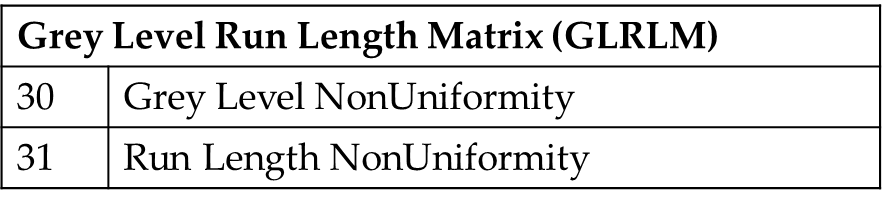

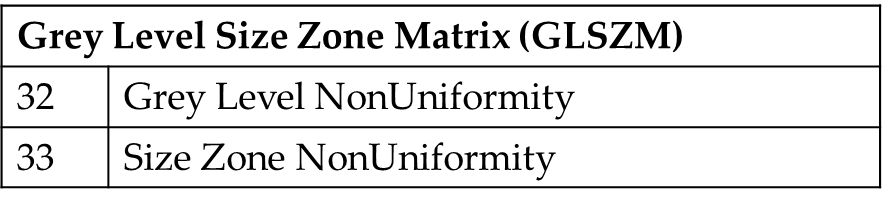

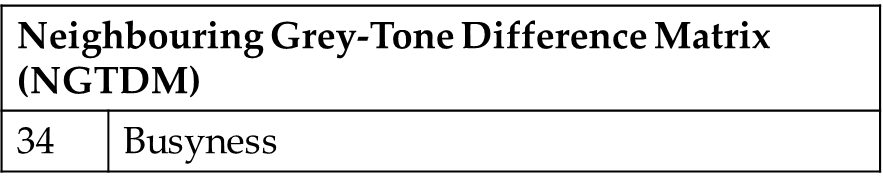

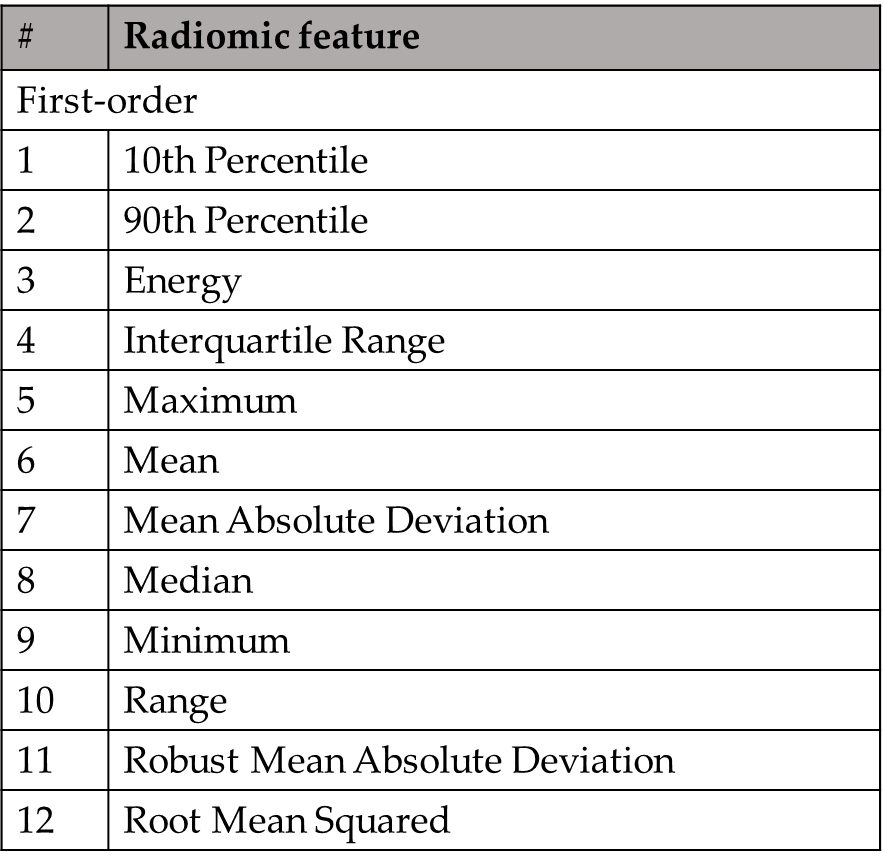

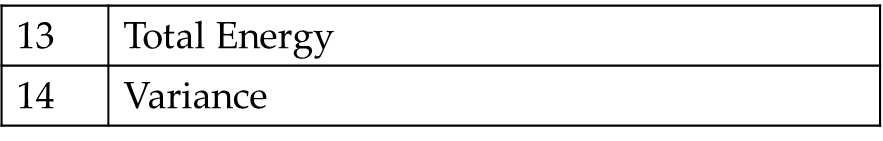

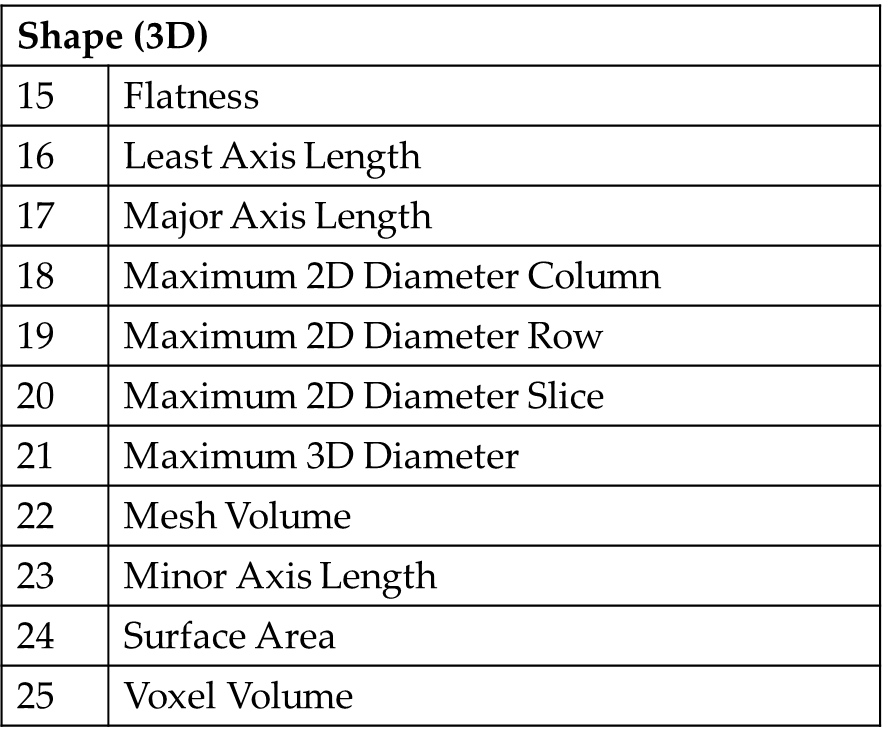

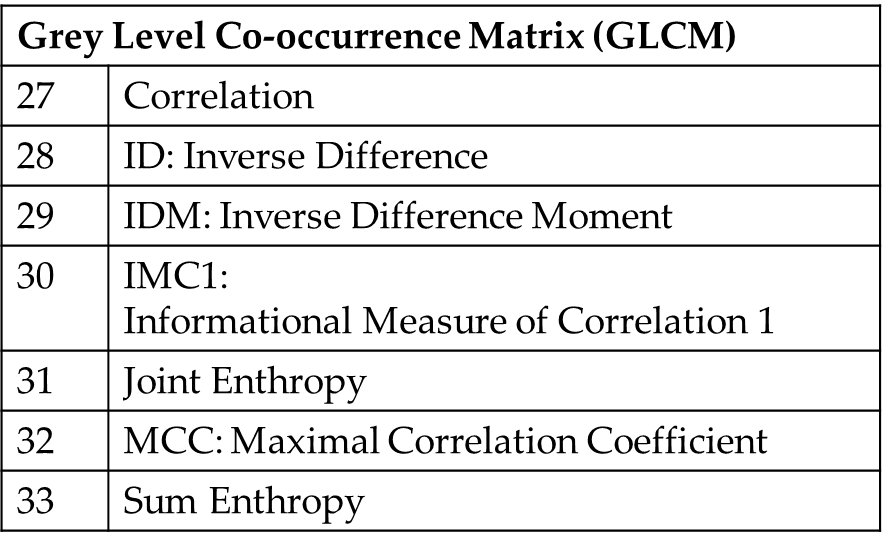

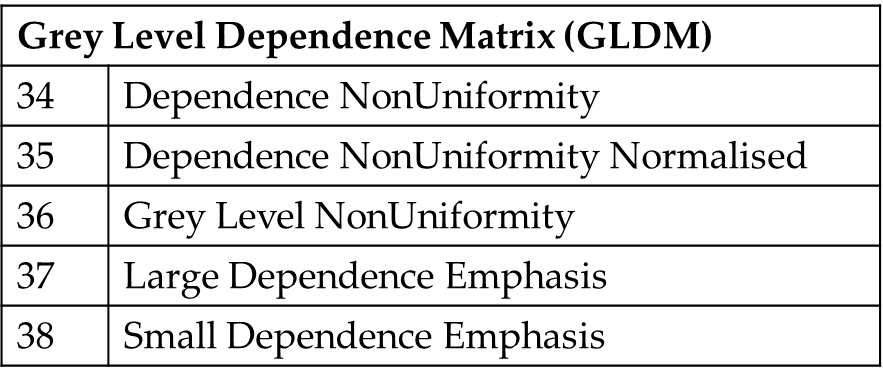

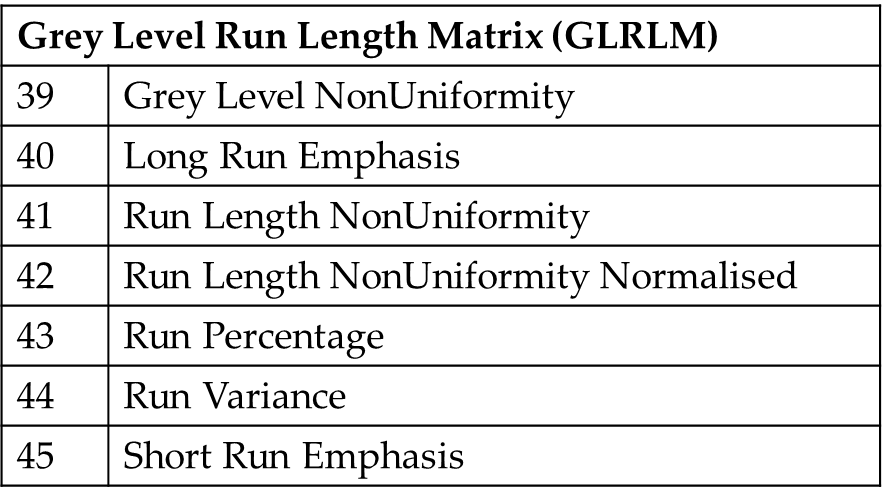

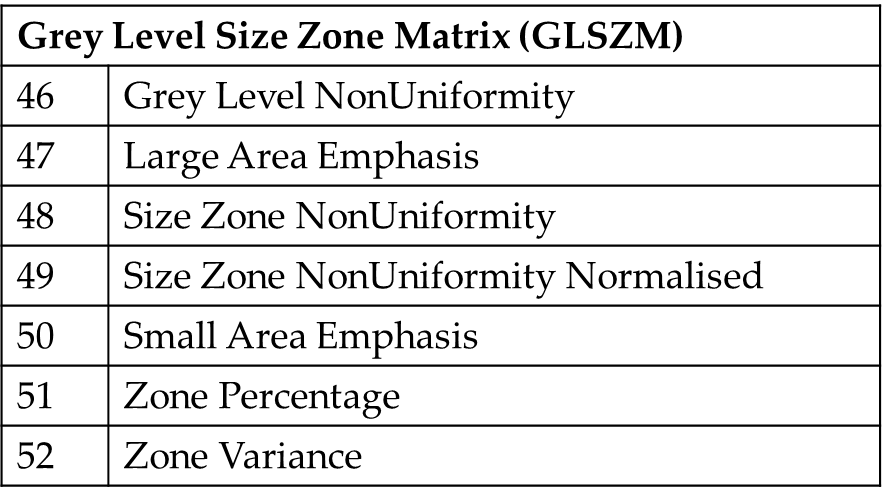

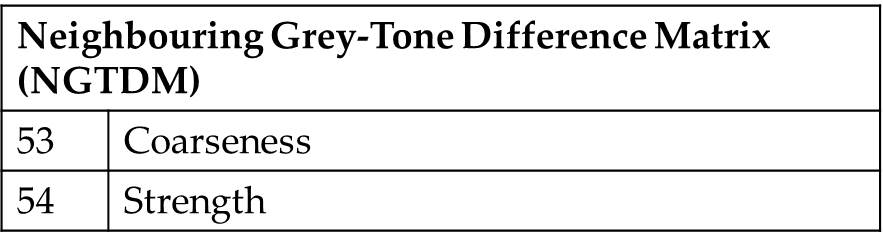


**Supplementary Table S4.** List of robust radiomic T2WI- and ADC-derived radiomic features used for delta-radiomics predictive modelling.

**References:**

1. Giganti F, Pecoraro M, Stavrinides V, et al (2020) Interobserver reproducibility of the PRECISE scoring system for prostate MRI on active surveillance: results from a two-centre pilot study. Eur Radiol 30:2082–2090. https://doi.org/10.1007/s00330-019-06557-2

2. Cattell R, Chen S, Huang C (2019) Robustness of radiomic features in magnetic resonance imaging: review and a phantom study. Vis Comput Ind Biomed Art 2:. https://doi.org/10.1186/s42492-019-0025-6

3. Scalco E, Belfatto A, Mastropietro A, et al (2020) T2w‐MRI signal normalization affects radiomics features reproducibility. Med Phys 47:1680–1691. https://doi.org/10.1002/mp.14038

4. Přibil J, Přibilová A, Frollo I (2019) Analysis of the influence of different settings of scan sequence parameters on vibration and noise generated in the open-air MRI scanning area. Sensors (Switzerland) 19:. https://doi.org/10.3390/s19194198
